# Cardiac metallothionein overexpression rescues diabetic cardiomyopathy in Akt2‐knockout mice

**DOI:** 10.1111/jcmm.16687

**Published:** 2021-05-30

**Authors:** Shan Huang, Jiqun Wang, Hongbo Men, Yi Tan, Qian Lin, Evelyne Gozal, Yang Zheng, Lu Cai

**Affiliations:** ^1^ Department of Pediatrics Pediatric Research Institute University of Louisville School of Medicine Louisville KY USA; ^2^ Department of Cardiovascular Disease The First Hospital of Jilin University Changchun China; ^3^ Department of Pharmacology and Toxicology University of Louisville Louisville KY USA; ^4^ Department of Radiation Oncology University of Louisville School of Medicine Louisville KY USA

**Keywords:** Akt2 knock out, diabetes, glucose metabolism, insulin resistance, metallothionein

## Abstract

To efficiently prevent diabetic cardiomyopathy (DCM), we have explored and confirmed that metallothionein (MT) prevents DCM by attenuating oxidative stress, and increasing expression of proteins associated with glucose metabolism. To determine whether Akt2 expression is critical to MT prevention of DCM, mice with either global Akt2 gene deletion (Akt2‐KO), or cardiomyocyte‐specific overexpressing MT gene (MT‐TG) or both combined (MT‐TG/Akt2‐KO) were used. Akt2‐KO mice exhibited symptoms of DCM (cardiac remodelling and dysfunction), and reduced expression of glycogen and glucose metabolism‐related proteins, despite an increase in total Akt (*t*‐Akt) phosphorylation. Cardiac MT overexpression in MT‐TG/Akt2‐KO mice prevented DCM and restored glucose metabolism‐related proteins expression and baseline *t*‐Akt phosphorylation. Furthermore, phosphorylation of ERK1/2 increased in the heart of MT‐TG/Akt2‐KO mice, compared with Akt2‐KO mice. As ERK1/2 has been implicated in the regulation of glucose transport and metabolism this increase could potentially underlie MT protective effect in MT‐TG/Akt2‐KO mice. Therefore, these results show that although our previous work has shown that MT preserving Akt2 activity is sufficient to prevent DCM, in the absence of Akt2 MT may stimulate alternative or downstream pathways protecting from DCM in a type 2 model of diabetes, and that this protection may be associated with the ERK activation pathway.

## 1 INTRODUCTION

Diabetic cardiomyopathy (DCM) is one of the dominant cardiovascular complications in diabetes.^1^ Studies of DCM pathogenesis have focused on oxidative stress, cardiomyocyte apoptosis, myocardial insulin resistance, endoplasmic reticulum stress, endothelial dysfunction, as well as mitochondrial dysfunction and autophagy.^2^, ^3^, ^4^ Nonetheless, the exact pathogenesis remains not well understood.

We have reported that zinc supplementation could significantly prevent cardiac functional and pathological anomalies in type 1 diabetic (T1D) mice, associated with the induction of cardiac metallothionein (MT).^5^ MTs are a group of ubiquitous and metal‐binding proteins. These unique structures determine MT's ability for metal binding and redox balancing.^6^ Both in vivo and in vitro studies have confirmed that MTs appeared to scavenge free radicals such as superoxide and hydroxyl radicals and therefore protected the DNA and cell structures from damage.^7^ So far, four mammalian MT (MT1, MT2, MT3 and MT4) isoforms have been identified. MT1 and MT2 are present in all types of soft tissues; therefore, these isoforms were more extensively investigated.^8^

We also reported that MT not only has antioxidant effect in diabetes, but also preserves the function of Akt2 and its downstream glucose metabolic pathways under diabetic conditions.^9^, ^10^ Protein kinase B (PKB/Akt) family have three isoforms. Among them, Akt2 is mainly involved in the insulin signalling pathway and is a vital molecule regulating glucose metabolism. Mice with global Akt2 gene deletion (Akt2‐KO) or people having Akt2 mutation gene have classic insulin resistance and type 2 diabetes (T2D) phenotype.^11^, ^12^, ^13^

Although it is established that both zinc and MT can provide protection from the development of DCM, affiliated with stimulation of Akt2 function and its downstream pathways in both T1D and T2D diabetes,^9^, ^10^ whether this protective effects fully depend on Akt2 remain unclear. Therefore, in this study, we used global Akt2‐KO mice with cardiomyocyte‐specifically overexpressing MT gene (MT‐TG/Akt2‐KO) to define whether these mice remain resistant to the development of DCM, that is whether the protection of MT against DCM exclusively depends on Akt2.

## 2 EXPERIMENTAL DESIGN AND METHODS

### 2.1 Animal models

Both male and female MT‐TG mice with FVB (friend virus B) background were used in this study. MT‐TG mice specifically overexpressed human MT2a in the cardiomyocytes^14^ and have been used in our previous studies.^9^, ^10^, ^15^, ^16^, ^17^ C57BL/6J background Akt2‐KO mice were originally purchased from the Jackson Laboratory (Bar Harbor, Maine) and then cross‐bred with FVB mice for more than 12 generations.^18^ FVB background Akt2‐KO mice displayed typical T2D features: insulin resistance (impaired glucose tolerance), hyperinsulinemia, and hyperglycaemia,^18^ consistent with previous reports.^11^, ^12^ Transgenic MT‐TG/Akt2‐KO mice were generated by cross‐breeding of FVB background Akt2‐KO mice with FVB background MT‐TG mice. Therefore, both male and female four lines of mice (FVB, Akt2‐KO, MT‐TG and MT‐TG/Akt2‐KO) were used for the present study. All mice were housed at 22°C with a 12:12‐hours light‐dark cycle with free access to rodent chow and tap water. Females and males were fed separately. Mice were intraperitoneally anaesthetized with Avertin (2,2,2‐tribromoethanol, 350 mg/kg) to collect hearts for weighing and performing pathological and biochemical examinations (at 24 weeks old). All animal‐related procedures were approved by Institutional Animal Care and Use Committee of University of Louisville. All mice were killed uniformly at 24 weeks.

### 2.2 Echocardiography

Echocardiography (Echo) was done three days before the mice were killed. Two dimensional and left ventricle M‐mode echocardiography was used to assess wall motion, chamber dimensions and cardiac function. A high‐resolution small animal imaging system (Vevo 770, VisualSonics, Canada), equipped with a high‐frequency ultrasound probe (RMV‐707B), was used to detect Avertin anaesthetized mice in the resting state by transthoracic echocardiography.

### 2.3 Histological staining

Sirius red staining: The staining was performed as described in our earlier publication.^19^ The proportion of fibrosis (collagen) in Sirius Red‐stained sections was then assessed using a computer‐assisted image analysis system. WGA staining: FITC coupled to wheat germ agglutinin (Invitrogen) staining was used to determine the size of cardiomyocytes. PAS staining: Cardiac tissue sections were subjected to Periodic Acid‐Schiff (PAS) for assessment of glycogen, as previously described.^20^

### 2.4 Western blot

Cardiac tissue was homogenized with RIPA lysis buffer to prepare the lysate. Protein samples were subjected to SDS‐PAGE and then transferred to PVDF membranes (Bio‐Rad, Hercules, CA). After blocking with 5% BSA (Bovine serum albumin), the membrane was incubated with the antibody. The primary antibodies used include Fibronectin (FN), TGF‐β, ICAM‐1, VCAM‐1, IL‐1β, all of which were purchased from Abcam, Cambridge, MA. Phospho (*p*)‐*t*‐Akt, total (*t*‐)Akt, *p*‐Akt1, Akt1, *p*‐Akt2, Akt2, *p*‐GSK‐3β, GSK‐3β, *p*‐GS, GS, *p*‐AS160, AS160, *p*‐ERK1/2, ERK1/2, hexokinase II (HK II), all of which were purchased from Cell Signaling Technology, Beverly, MA. COL1A1, β‐Actin, all of which were purchased from Santa Cruz Biotech. Inc 3‐NT was purchased from Millipore. 4‐HNE was purchased from Alpha Diagnostic. Inc *p*‐PFKFB2, PFKFB2 and glycogen phosphorylase (GP) were purchased from Thermo Fisher Scientific, Waltham, MA. Antibody against MT was purchased from DakoCytomation, Santa Clara, CA. The ratios of total protein of Akt2, *t*‐Akt, Akt1, GSK‐3β, GS, AS160, PFKFB2 and ERK1/2 to β‐Actin are in the supplementary figures.

### 2.5 Statistical analysis

Data are expressed as mean ± standard deviation (SD). Intergroup differences were assessed using a one‐way ANOVA, followed by post hoc pairwise repetitive comparisons with Turkey test using GraphPad Prism version 8.0.2.

## 3 RESULTS

### 3.1 The preventive effects of overexpressed cardiac MT on cardiac dysfunction and pathological abnormalities in Akt2‐KO mice

**FIGURE 1:**
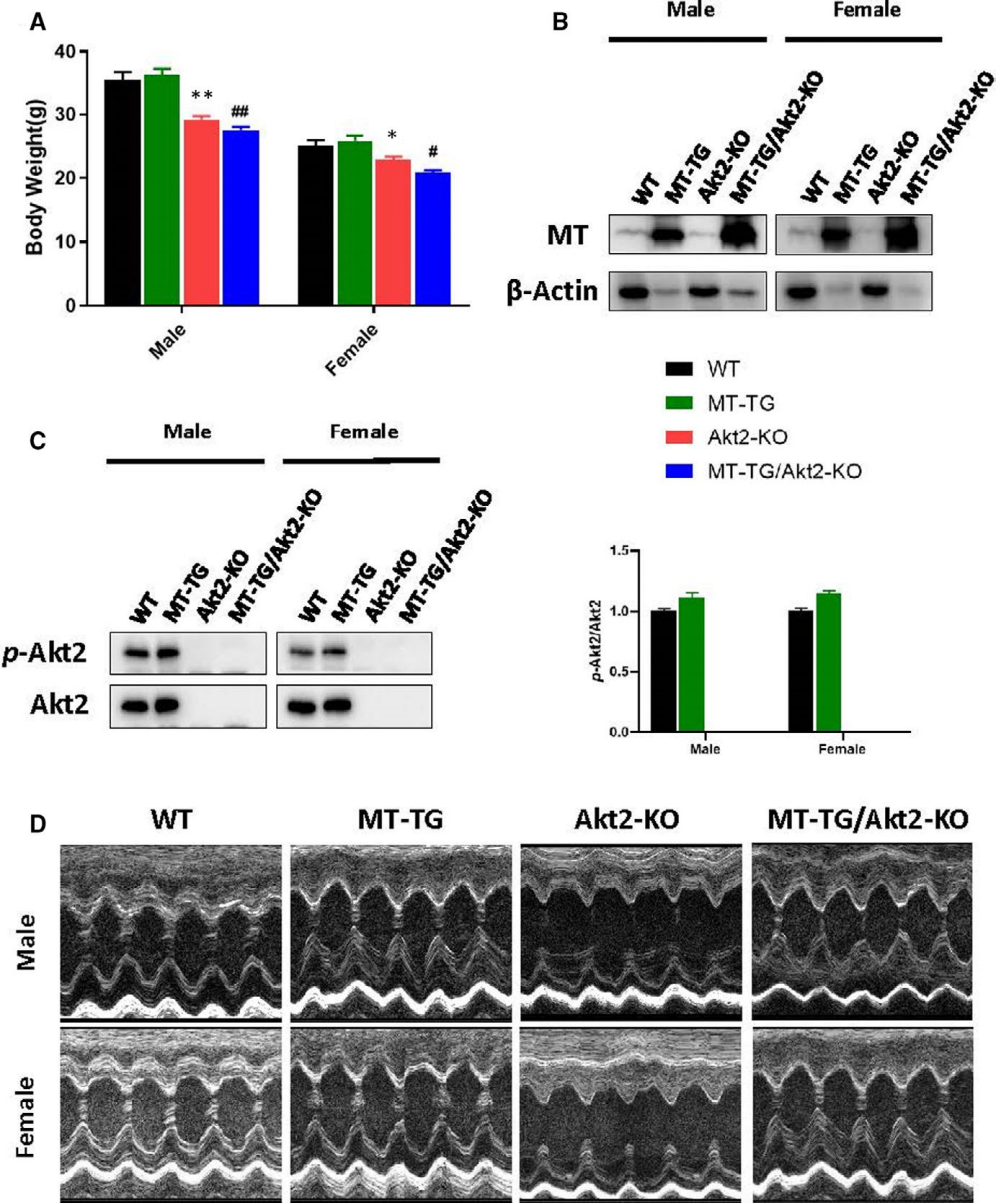
Bodyweight, genetic identification and echocardiographic images of mice. Bodyweight (A) was measured. MT (B) and Akt2 (C) was detected by Western blot. Left vol short‐axis M‐mode echocardiography (D) was measured. Data are presented as mean ± SD (WT: n = 9 in male; n = 6 in female; MT‐TG: n = 8 in male; n = 5 in female; Akt2‐KO: n = 8 in male; n = 8 in female; MT‐TG/Akt2‐KO: n = 9 in male; n = 9 in female). Quantitation plots are expressed as fold WT control (Akt2). *P <.05 vs the WT; **P <.01 vs the WT; #*P* <.05 vs MT‐TG; ##*P* <.01 vs MT‐TG

There was a significant decrease in the bodyweight of Akt2‐KO and MT‐TG/Akt2‐KO mice compared to the WT mice at 24 weeks, although there was no bodyweight change in MT‐TG mice compared to WT control (Figure 1A). This phenomenon of decreased bodyweight of Akt2‐KO and MT‐TG/Akt2‐KO is more obvious in male mice than females. Next, we confirmed the genotypes of these mice. Western blots of MT or Akt2 and Akt2 phosphorylation (*p*‐Akt2) revealed that MT expression was significantly higher in the MT‐TG and MT‐TG/Akt2‐KO mice than control mice (Figure 1B), and there was no expression of Akt2 or *p*‐Akt2 in Akt2‐KO and MT‐TG/Akt2‐KO mice (Figure 1C; Figure S1 for detail). There was no significant difference in the level of *p*‐Akt2 between MT‐TG and WT mice, regardless of male and female (Figure 1C). These results confirm the correct genotypes of these transgenic and cross‐bred double transgenic mice.

Cardiac function was examined for these mice before killing with echocardiography (Echo), for which the representative images are provided in the Figure 1D. As illustrated in the table below, Echo analysis revealed that all structural changes are increased in Akt2‐KO mice and these conditions were more serious during systole as presented in the table below. All the observations described above are worse in male mice. However, all these structural abnormalities seen in Akt2‐KO mice were abolished in MT‐TG/Akt2‐KO mice.

**FIGURE 2:**
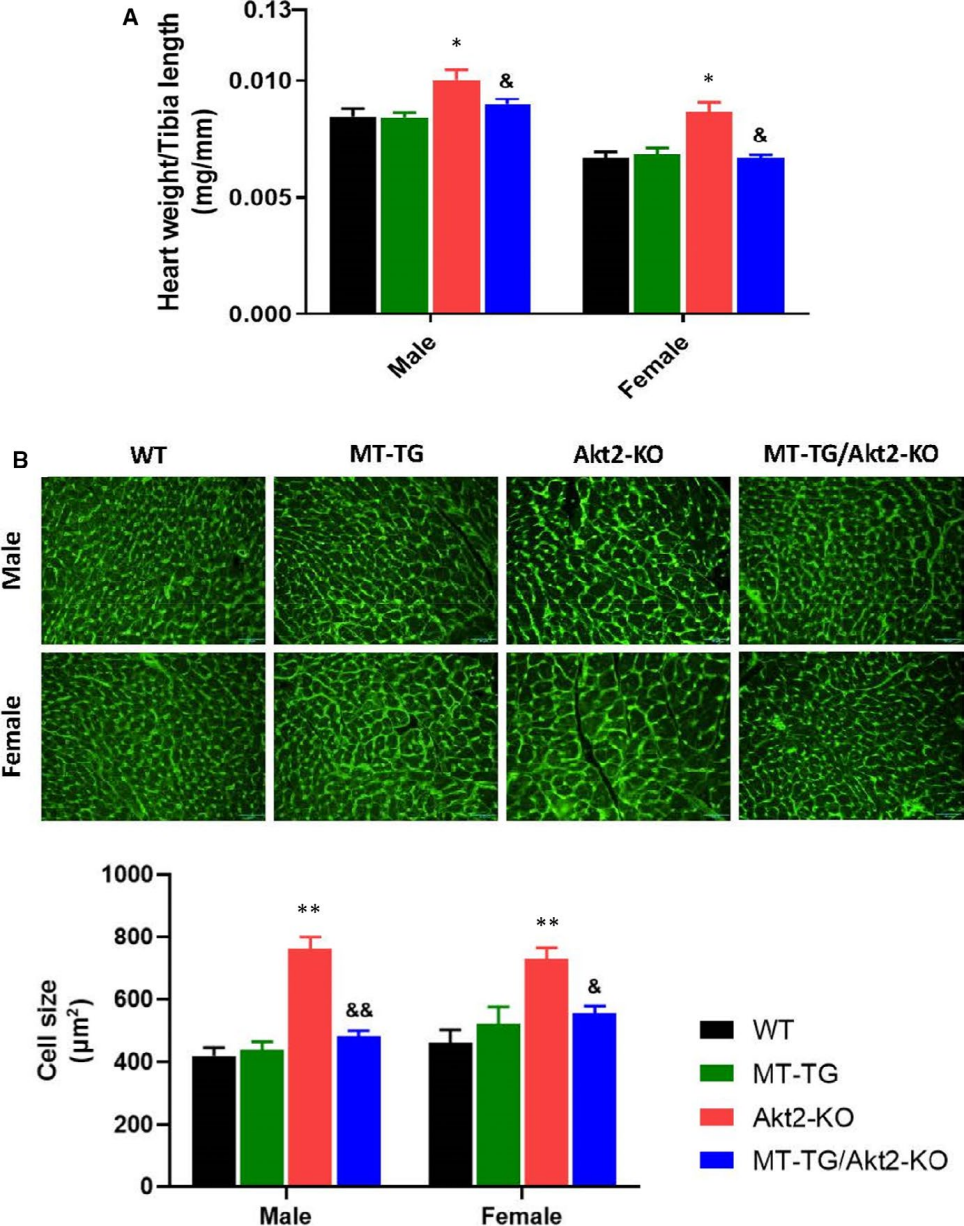
Index for cardiac hypertrophy. Ratio of the heart weight to tibia length (A) was calculated at the end of study. Wheat germ agglutinin (green)‐stained cardiac cross‐sections with quantification of mean cardiomyocyte cross‐sectional area relative fold change (B). Data are presented as mean ± SD (WT: n = 9 in male; n = 6 in female; MT‐TG: n = 8 in male; n = 5 in female; Akt2‐KO: n = 8 in male; n = 8 in female; MT‐TG/Akt2‐KO: n = 9 in male; n = 9 in female). *P <.05 vs the WT; **P <.01 vs the WT; &*P* <.05 vs Akt2‐KO; &&*P* <.01 vs Akt2‐KO

In the Akt2‐KO mice, the cardiac ejection fraction (EF%) and the fractional shortening (FS%) were both significantly reduced, suggesting that cardiac function was significantly affected (Table 1). These conditions were all improved by cardiac overexpressed MT in MT‐TG/Akt2‐KO. Echo measurements also showed that the corrected LV mass also increased (Table 1). In support of Echo observation, Akt2‐KO mice showed a significant increase in the ratio of heart weight to tibia length compared with that of the WT mice (Figure 2A). However, this increased heart to tibia length ratio in Akt2‐KO mice was not seen in the MT‐TG/Akt2‐KO group, indicating that hearts in Akt2‐KO mice were hypertrophic. The echo analysis suggests the high expression of MT in the heart can obviously prevent the cardiac hypertrophy (LV vol). At the cellular level, WGA staining also showed cardiac cell hypertrophy in Akt2‐KO mice, but not in MT‐TG/Akt2‐KO mice compared with WT mice or MT‐TG mice (Figure 2B).

**TABLE 1:** Cardiac echocardiographic measurements of structural and functional cardiac changes

		WT	MT‐TG	Akt2‐KO	MT‐TG/Akt2‐KO
Male	HR (beats/min)	482 ± 36	493 ± 42	472 ± 43	488 ± 44
	IVS;d (mm)	0.70 ± 0.04	0.72 ± 0.03	0.68 ± 0.05	0.69 ± 0.04
	IVS;s (mm)	1.03 ± 0.07	1.04 ± 0.04	0.94 ± 0.07*	1.01 ± 0.07
	LVID;d (mm)	3.76 ± 0.13	3.94 ± 0.17	4.33 ± 0.24**	3.95 ± 0.18^†^
	LVID;s (mm)	2.12 ± 0.13	2.19 ± 0.14	2.81 ± 0.22**	2.25 ± 0.14^‡^
	LVPW;d (mm)	0.80 ± 0.06	0.83 ± 0.06	0.75 ± 0.03	0.79 ± 0.05
	LVPW;s (mm)	1.17 ± 0.07	1.21 ± 0.06	1.08 ± 0.06*	1.11 ± 0.07
	LV Vol;d (mm)	60.55 ± 5.09	67.92 ± 6.95	84.68 ± 11.52**	68.30 ± 7.27^†^
	LV Vol;s (mm)	14.91 ± 2.39	16.06 ± 2.53	29.94 ± 5.46**	17.25 ± 2.79^‡^
	EF,%	75.39 ± 3.09	76.44 ± 1.84	64.52 ± 5.96**	74.84 ± 1.8^‡^
	FS,%	43.54 ± 2.76	44.6 ± 1.67	35.15 ± 4.48**	43.14 ± 1.54^‡^
	LV Mass (mg)	96.9 ± 5.78	109.9 ± 11.11	116 ± 13.25*	104.1 ± 13.93
	LV Mass Corrected (mg)	77.52 ± 4.63	87.93 ± 8.89	92.8 ± 10.6*	83.25 ± 11.14

		WT	MT‐TG	Akt2‐KO	MT‐TG/Akt2‐KO
Female	HR (beats/min)	472 ± 43	485 ± 39	474 ± 37	478 ± 28
	IVS;d (mm)	0.66 ± 0.04	0.67 ± 0.05	0.63 ± 0.04	0.64 ± 0.03
	IVS;s (mm)	0.98 ± 0.04	0.96 ± 0.1	0.90 ± 0.07	0.93 ± 0.08
	LVID;d (mm)	3.74 ± 0.08	3.65 ± 0.25	3.84 ± 0.21	3.75 ± 0.2
	LVID;s (mm)	2.09 ± 0.08	1.98 ± 0.21	2.43 ± 0.21*	2.12 ± 0.2^†^
	LVPW;d (mm)	0.73 ± 0.04	0.76 ± 0.06	0.72 ± 0.08	0.73 ± 0.05
	LVPW;s (mm)	1.08 ± 0.12	1.11 ± 0.1	0.96 ± 0.09*	1.01 ± 0.07
	LV Vol;d (mm)	59.74 ± 3.2	56.38 ± 9.1	63.72 ± 8.3	60.13 ± 7.74
	LV Vol;s (mm)	14.45 ± 1.38	12.63 ± 3.26	20.98 ± 4.58*	15.01 ± 3.5^†^
	EF,%	75.81 ± 1.96	77.75 ± 3.69	67.21 ± 4.7**	75.23 ± 3.6^‡^
	FS,%	43.86 ± 1.8	45.7 ± 3.56	36.8 ± 3.62**	43.41 ± 3.22^‡^
	LV Mass(mg)	87.07 ± 6.33	86.52 ± 12.38	87.97 ± 11.78	85.3 ± 7.01
	LV Mass Corrected (mg)	69.66 ± 5.06	69.21 ± 9.9	70.38 ± 9.42	68.24 ± 5.6

Note

Data are presented as mean ± SD (WT: n = 9 in male; n = 6 in female; MT‐TG: n = 8 in male; n = 5 in female; Akt2‐KO: n = 8 in male; n = 8 in female; MT‐TG/Akt2‐KO: n = 9 in male; n = 9 in female).

Abbreviations: EF, ejection fraction; FS, fractional shortening; HR, heart rates; IVS, Interventricular septum; LV Vol, LV volume; LVID, d, Left ventricular (LV) end‐diastolic diameter; LVID, s, LV end‐systolic diameter; LVPW, LV posterior wall.

* *P* <.05 vs the wild type (WT).

** *P* <.01 vs the WT.

^†^ *P* <.05 vs Akt2‐KO.

^‡^ *P* <.01 vs Akt2‐KO.

**FIGURE 3:**
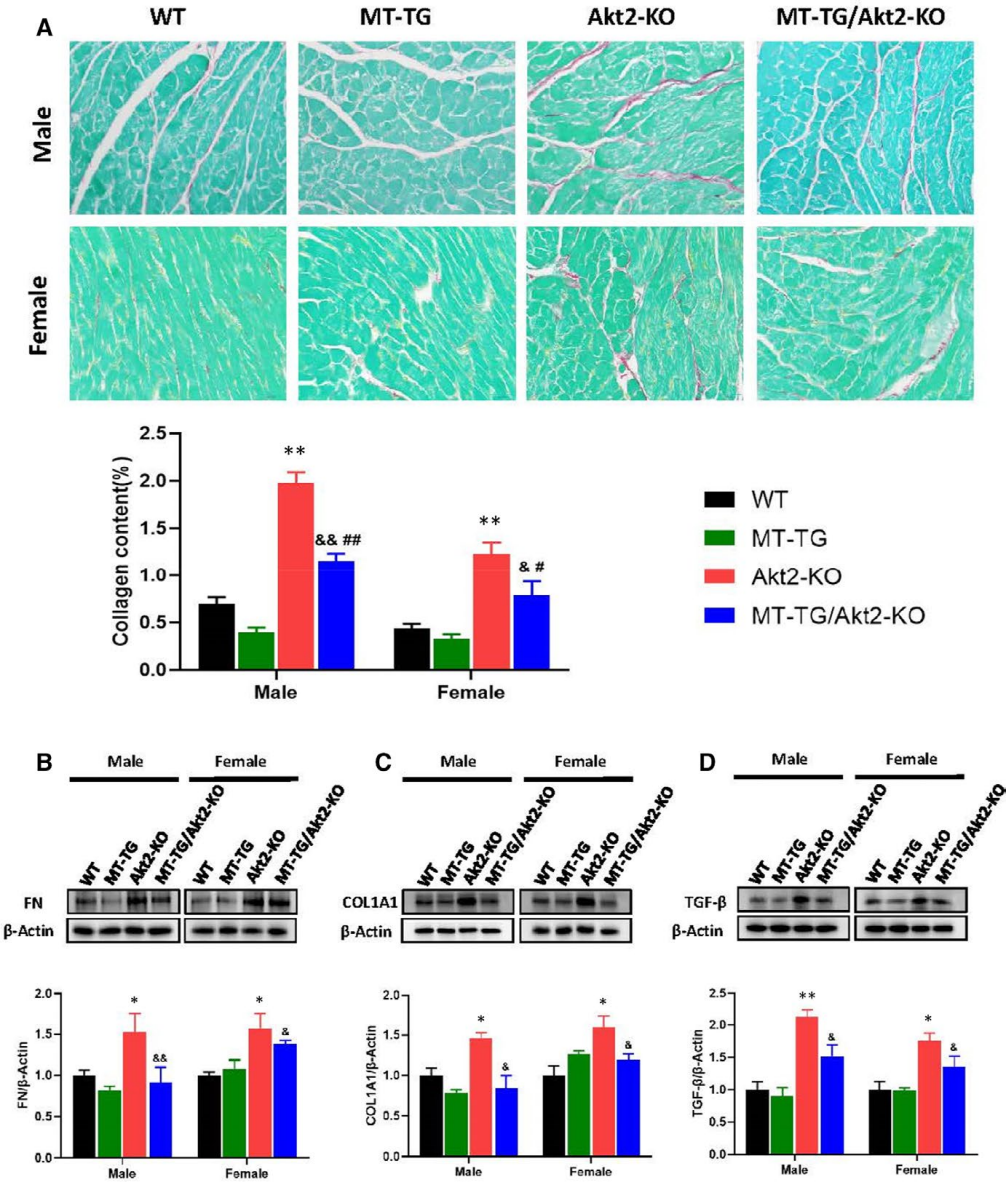
Preventive effects of MT‐TG against cardiac fibrosis in Akt2‐KO mice. Cardiac fibrotic remodelling was examined with Sirius red staining (A). Sirius red positive staining was semi‐quantified using a computer imaging analysis system (Image J). Cardiac expression of fibronectin (FN) (B), COL1A1 (C) and TGF‐β (D) as index of fibrotic mediators was detected by Western blot. Data are presented as mean ± SD (WT: n = 9 in male; n = 6 in female; MT‐TG: n = 8 in male; n = 5 in female; Akt2‐KO: n = 8 in male; n = 8 in female; MT‐TG/Akt2‐KO: n = 9 in male; n = 9 in female). Quantitation plots are expressed as fold WT control. *P <.05 vs the WT; **P <.01 vs the WT; &*P* <.05 vs Akt2‐KO; &&*P* <.01 vs Akt2‐KO; #*P* <.05 vs MT‐TG; ##*P* <.01 vs MT‐TG

The Sirius red staining analysis confirmed a large accumulation of collagen in the heart of Akt2‐KO mice, but not MT‐TG/Akt2‐KO mice (Figure 3A). The fibrotic effect in the heart is more severe in male mice compared with females. Western blotting of cardiac tissues also showed the fibrotic response, reflected by increased expression levels of fibronectin (FN), collagen 1A1 (COL1A1) and transforming growth factor‐β (TGF‐β) in Akt2‐KO mice but not in MT‐TG/Akt2‐KO mice compared with WT or MT‐TG mice (Figure 3B‐D). The above results show that cardiac overexpression of MT can reduce cardiac fibrosis in Akt2‐KO mice. When comparing females with males, the accumulation of collagen (Figure 3A) and TGF‐β (Figure 3D) was higher in males but there was no significant difference between males and females in the accumulation of FN and COL1A1.

### 3.2 The preventive effects of overexpressed cardiac MT on cardiac inflammation and oxidative stress and damage in Akt2‐KO mice

**FIGURE 4:**
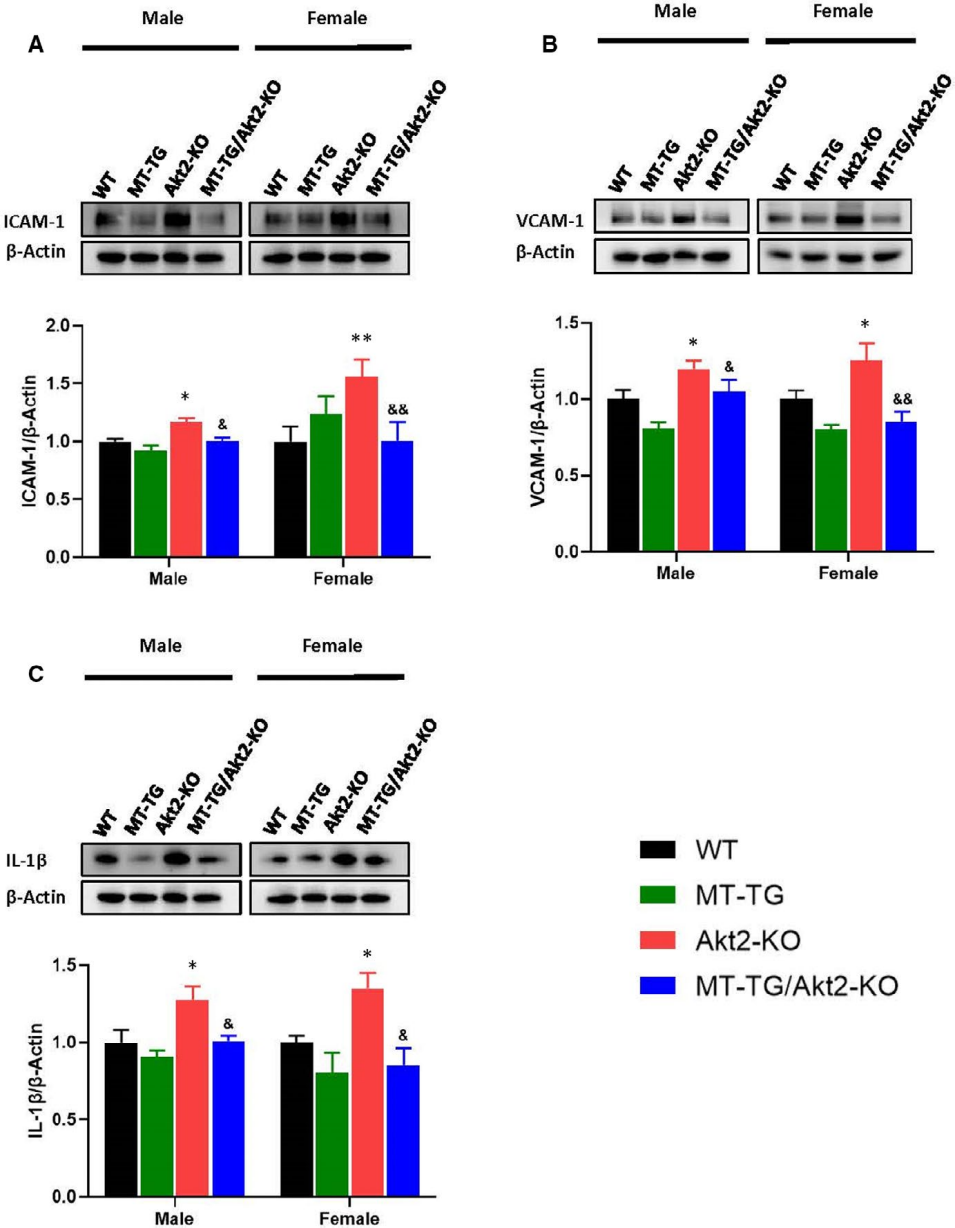
Preventive effects of MT‐TG on cardiac inflammation damages in Akt2‐KO mice. Cardiac expression of ICAM‐1 (A), VCAM‐1 (B) and IL‐1β (C), as indexes of inflammation, were detected by a Western blot. Figures A and C are the same gel and membrane using the same β‐Actin. Data are presented as mean ± SD (WT: n = 9 in male; n = 6 in female; MT‐TG: n = 8 in male; n = 5 in female; Akt2‐KO: n = 8 in male; n = 8 in female; MT‐TG/Akt2‐KO: n = 9 in male; n = 9 in female). Quantitation plots are expressed as fold WT control. *P <.05 vs the WT; **P <.01 vs the WT; &*P* <.05 vs Akt2‐KO; &&*P* <.01 vs Akt2‐KO

As indicators of inflammation, the expression and accumulation of ICAM‐1, VCAM‐1 and IL‐1β were examined and showed a significant increase in these inflammatory markers in Akt2‐KO mice, but not in the MT‐TG/Akt2‐KO mice, compared to control or MT‐TG mice (Figure 4A‐C). Compared with male mice, ICAM‐1 expression was significantly higher in female Akt2‐KO mice but there was no significant difference for VCAM‐1 and IL‐1β expression. MT alleviated inflammatory responses in MT‐TG/Akt2‐KO significantly better in females than in males.

**FIGURE 5:**
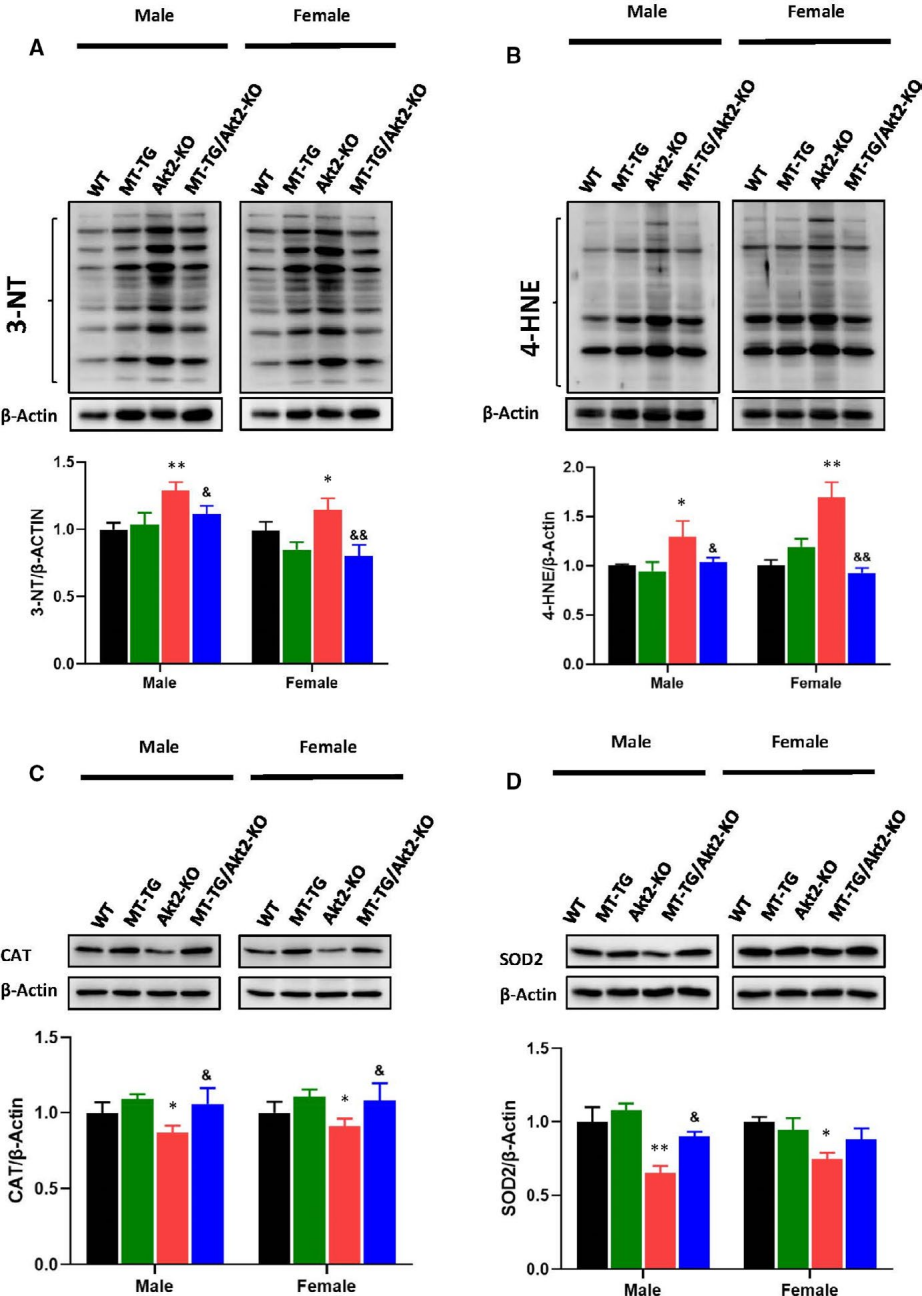
Preventive effects of MT‐TG on cardiac oxidative damage in Akt2‐KO mice. Cardiac expression of 3‐NT (A), 4‐HNE (B), as indexes of oxidative stress damages and of antioxidant enzymes CAT(C), SOD2(D), were detected by Western blot. Data are presented as mean ± SD (WT: n = 9 in male; n = 6 in female; MT‐TG: n = 8 in male; n = 5 in female; Akt2‐KO: n = 8 in male; n = 8 in female; MT‐TG/Akt2‐KO: n = 9 in male; n = 9 in female). Quantitation plots are expressed as fold WT control. *P <.05 vs the WT; **P <.01 vs the WT; &*P* <.05 vs Akt2‐KO; &&*P* <.01 vs Akt2‐KO

Oxidative damage was assessed by examining the levels of 3‐nitrotyrosine (3‐NT) for protein nitrosylation and 4‐Hydroxynonenal (4‐HNE) for lipid peroxidation indexes (Figure 5A,B). Immunoblotting revealed significant increases in 3‐NT and 4‐HNE expression in Akt2‐KO mice, but not in MT‐TG/Akt2‐KO mice, compared with WT and MT‐TG mice, suggesting that cardiac overexpression of MT can almost completely prevent Akt2‐KO‐associated increases in 3‐NT and 4‐HNE accumulation in the heart. Gender did not contribute any additional effect on the changes in nitrosative damages, but in female mice, the oxidative damage assessed by 4‐HNE, was enhanced, compared with male mice (Figure 5A,B). As oxidative stress could be caused by either increased reactive oxygen or nitrogen species or decreased antioxidants, antioxidant enzymes levels were examined. Catalase (CAT) and superoxide dismutase (SOD2) expression were not significantly changed in MT‐TG mice compared with WT mice, but significantly decreased in Akt2‐KO mice, which was prevented by cardiac overexpression of MT in MT‐TG/Akt2‐KO mice (Figure 5C,D). These results further suggest that MT can protect against oxidative damage caused by Akt2 deletion.

### 3.3 The protective effects of overexpressed cardiac MT on glucose metabolism‐related molecules in Akt2‐KO mice

**FIGURE 6:**
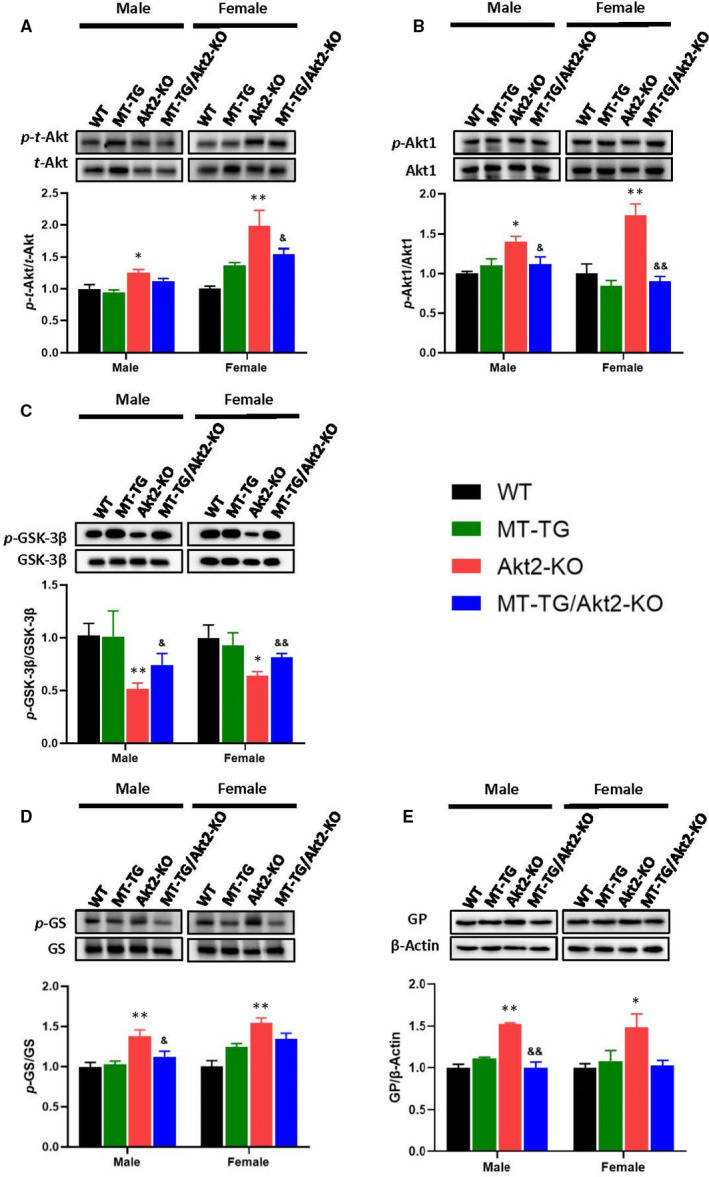
Effect of MT‐TG on the cardiac Akt2‐mediated glucose metabolism in Akt2‐KO mice. Cardiac expression and its phosphorylation of *t*‐Akt (A), Akt1 (B), GSK‐3β (C), GS (D) and GP (E) were detected by immunoblotting. Data are presented as mean ± SD (WT: n = 9 in male; n = 6 in female; MT‐TG: n = 8 in male; n = 5 in female; Akt2‐KO: n = 8 in male; n = 8 in female; MT‐TG/Akt2‐KO: n = 9 in male; n = 9 in female). Quantitation plots are expressed as fold WT control. *P <.05 vs the WT; **P <.01 vs the WT; &*P* <.05 vs Akt2‐KO; &&*P* <.01 vs Akt2‐KO

In order to explore whether Akt activation could play a role in cardiac MT‐mediated DCM protection in Akt2‐KO mice, we examined the expression and phosphorylation levels of total Akt (*t*‐Akt) and of Akt1, the most prominent Akt isoform, as well as of Akt2 downstream proteins involved in regulating glucose metabolism. The phosphorylation level of *t*‐Akt encompassing all Akt isoforms was increased in Akt2‐KO mice but not in MT‐TG/Akt2‐KO mice compared with WT mice or MT‐TG male mice. In female mice, the phosphorylation level in MT‐TG/Akt2‐KO was significantly lower than that in Akt2‐KO mice (Figure 6A; Figure S2). This suggested that other isoforms of Akt may play compensative roles in Akt2‐KO mice. Akt1 phosphorylation was indeed significantly elevated in Akt2‐KO mice compared with WT mice, particularly in females (Figure 6B; Figure S3). Akt1 phosphorylation was not changed in male MT‐TG mice, but trended lower (*P* > .05) in female MT‐TG mice compared with WT mice. However, Akt1 phosphorylation levels in female MT‐TG/Akt2‐KO mice were not further increased, compared with Akt2‐KO mice, and even comparable to MT‐TG mice (Figure 6B). These findings suggest that the lack of Akt2 expression in Akt2‐KO mice may be compensated by increased expression of other Akt isoforms which do not appear to play a role in the cardiac protective effect of MT in MT‐TG/Akt2‐KO.

Akt2 has been implicated in cardiac glucose metabolism. Therefore, we examined Akt2 downstream molecules that involve glucose and glycogen metabolism. We investigated cardiac glycogen synthesis and metabolism pathway enzymes, including phosphorylation of glycogen synthase kinase −3β (GSK‐3β), glycogen synthase (GS) and expression of glycogen phosphorylase (GP) (Figure 6C‐E; Figures S4 and S5). Western blots showed significantly decreased GSK‐3β phosphorylation and increased GS phosphorylation in Akt2‐KO mice, however, MT overexpression in the heart of Akt2‐KO mice (MT‐TG/Akt2‐KO) significantly prevented these effects. Cardiac MT overexpression in MT‐TG mice did not significantly affect GSK‐3β and GS phosphorylation level (the phosphorylation of GS in the MT‐TG female mice was slightly higher than the WT mice but did not reached statistical difference). The expression of GP in both male and female Akt2‐KO mice was significantly higher than WT mice, but not in MT‐TG/Akt2‐KO mice.

**FIGURE 7:**
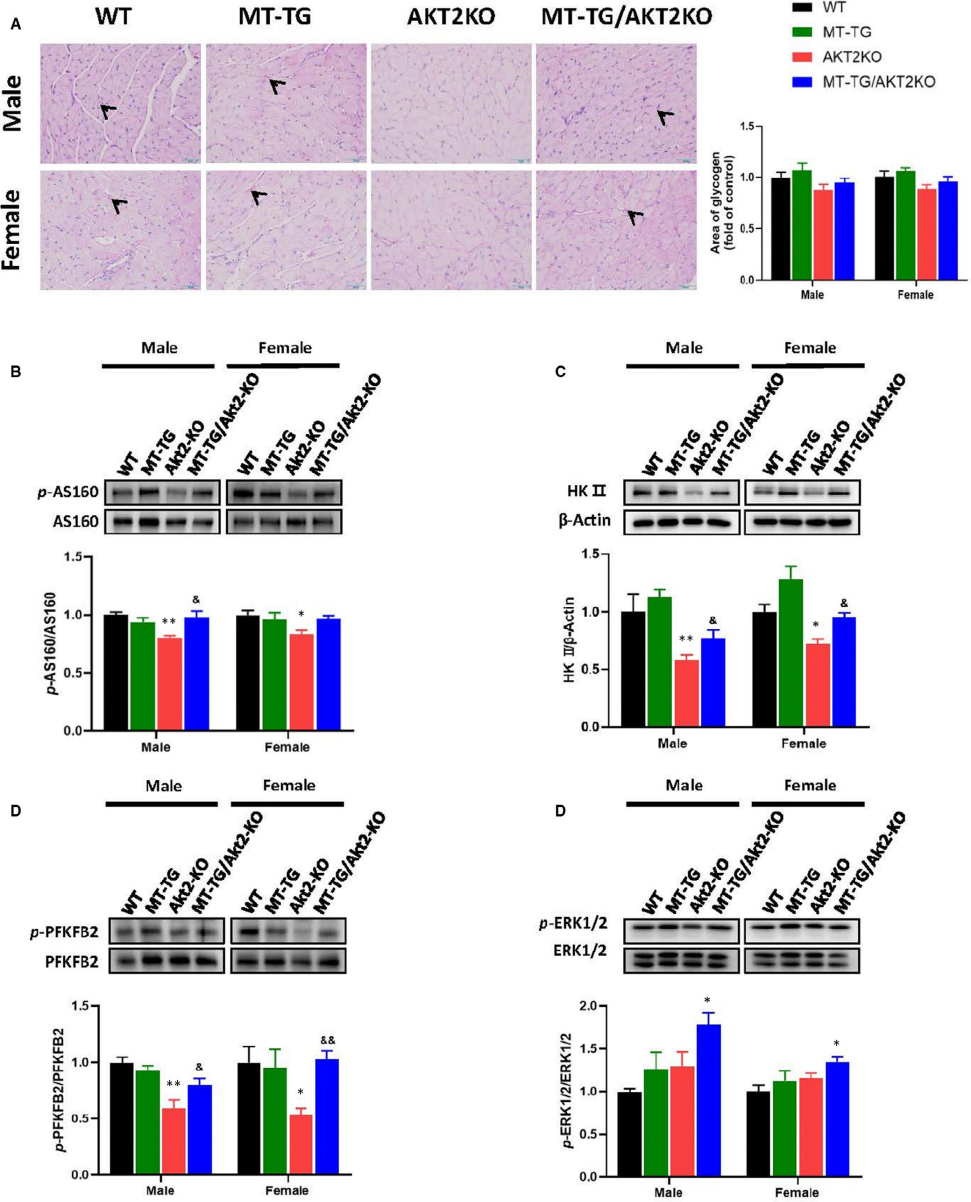
Effect of MT‐TG on cardiac Akt2‐mediated glucose metabolism in Akt2‐KO mice. Heart PAS staining (A), red spots mean glycogen. Cardiac expression and its phosphorylation of AS160 (B), HKII (C), PFKFB2 (D) and ERK1/2 (E) were detected by Western blot. Data are presented as mean ± SD (WT: n = 9 in male; n = 6 in female; MT‐TG: n = 8 in male; n = 5 in female; Akt2‐KO: n = 8 in male; n = 8 in female; MT‐TG/Akt2‐KO: n = 9 in male; n = 9 in female). Quantitation plots are expressed as fold WT control. *P <.05 vs the WT; **P <.01 vs the WT; &*P* <.05 vs Akt2‐KO; &&*P* <.01 vs Akt2‐KO

Positive spots of PAS staining were seen in the WT mice, but were almost absent in the Akt2‐KO mice (Figure 7A). Some spots could be detected in the MT‐TG/Akt2‐KO mice staining. Overall, these data show that glycogen metabolism is reduced in Akt2‐KO mice by either glycogen synthesis pathway or glycogen phosphorylation pathway and that increased cardiac MT expression can restore these pathways.

To examine Akt‐dependent glucose transport pathways, we examined the phosphorylation of Akt substrate of 160 kD (AS160), a regulator of glucose transporter 4 (GLUT4). AS 160 phosphorylation was significantly reduced in Akt2‐KO mice compared with WT mice, but was restored in MT‐TG/Akt2‐KO mice (Figure 7B; Figure S6). Akt2 is a vital protein that regulates the glycolysis.^21^ The expression of the glycolytic enzyme hexokinase II (HK II) and the phosphorylation of 6‐Phosphofructo‐2‐Kinase/Fructose‐2,6‐Biphosphatase 2) (PFKFB2) were also significantly reduced in Akt2‐KO mice, and cardiac MT overexpression completely or partially prevented these changes in MT‐TG/Akt2‐KO mice compared with Akt2‐KO mice (Figure 7C,D; Figure S7).

Our previous study confirmed that cardiac overexpression of MT in a type 1 diabetic model could promote the phosphorylation of Akt2 and of its down downstream molecules.^9^ As we found that despite the deletion of Akt2, MT still improved downstream glucose metabolism‐associated molecules in Akt2‐KO mice, it is reasonable to infer that MT likely compensate the regulation of these glucose metabolism‐associated molecules through Akt2‐independent pathways. It was reported that the ERK pathway plays a role in GSK‐3β inactivation, glucose transfer and glycolysis.^24^, ^25^, ^26^, ^27^, ^28^, ^29^ We therefore examined the phosphorylation of ERK1/2 by Western blot, which showed that ERK1/2 phosphorylation was not significantly altered in Akt2‐KO female mice compared with WT mice and only slightly higher in the male mice, but was significantly elevated in MT‐TG/Akt2‐KO mice compared to Akt2‐KO mice (Figure 7E; Figure S8). These results suggest that cardiac overexpression of MT may compensate for Akt2‐mediated glucose metabolism via activating ERK pathway in Akt2‐KO mice.

## 4 DISCUSSION

MT as a metal‐binding protein plays an important role in the regulation of intracellular zinc ion concentration,^22^ and zinc supplementation has now been shown to be beneficial in the treatment of diabetes.^23^, ^24^, ^25^ Numerous studies have shown that zinc provides important protective effects on hearts. Zinc deficiency increases coronary artery occlusion and cardiac lipid peroxide levels.^26^ Diabetics with lower serum zinc concentrations have a higher risk of cardiovascular disease compared with patients with normal or higher serum zinc concentrations.^27^ Our previous studies have confirmed that dietary zinc supplementation improves a range of functional and pathological changes resulting from Akt2 deletion.^18^ The concentration of cytoplasmic zinc also increased when MT protein expression increased.^28^ We speculate that the protective effect by cardiac overexpression of MT on Akt2‐KO mice may be related to increased zinc content.

In addition, MT is an important antioxidant.^29^ Persistent hyperglycaemia leads to the generation of advanced glycation end products, which causes oxidative damage, hypoxia and metabolic disorders.^1^, ^9^, ^13^ Metabolic disorders occur in the diabetic heart and activation of multiple systems can result in oxidative stress.^1^, ^30^ The administration of antioxidant stress drugs reduces the complications of diabetes remarkably.^1^, ^9^, ^13^ Our previous study has demonstrated that in T1D, cardiac MT overexpression in MT‐TG mice without deletion of Akt2 was able to inhibit the expression of Trb3 thus preserving the phosphorylation of Akt2 to regulate its downstream proteins even under diabetic condition.^9^ Trb3 is thought to be up‐regulated in response to oxidative stress, and then bind to Akt2 for accelerating its degradation via autophagy.^31^ In the present study with Akt2‐KO mice, we also found that cardiac overexpression of MT was able to preserve almost normal levels of Akt2 downstream glucose transport and metabolism‐associated molecule expression and function, and prevent oxidative damage in MT‐TG/Akt2‐KO mice, therefore, the MT prevention of DCM in Akt2‐KO mice could not be mediated by its preservation of Akt2 expression and function through suppressing Trb3 as Akt2 negatively regulator, so alternative mechanisms must be implicated.

**FIGURE 8:**
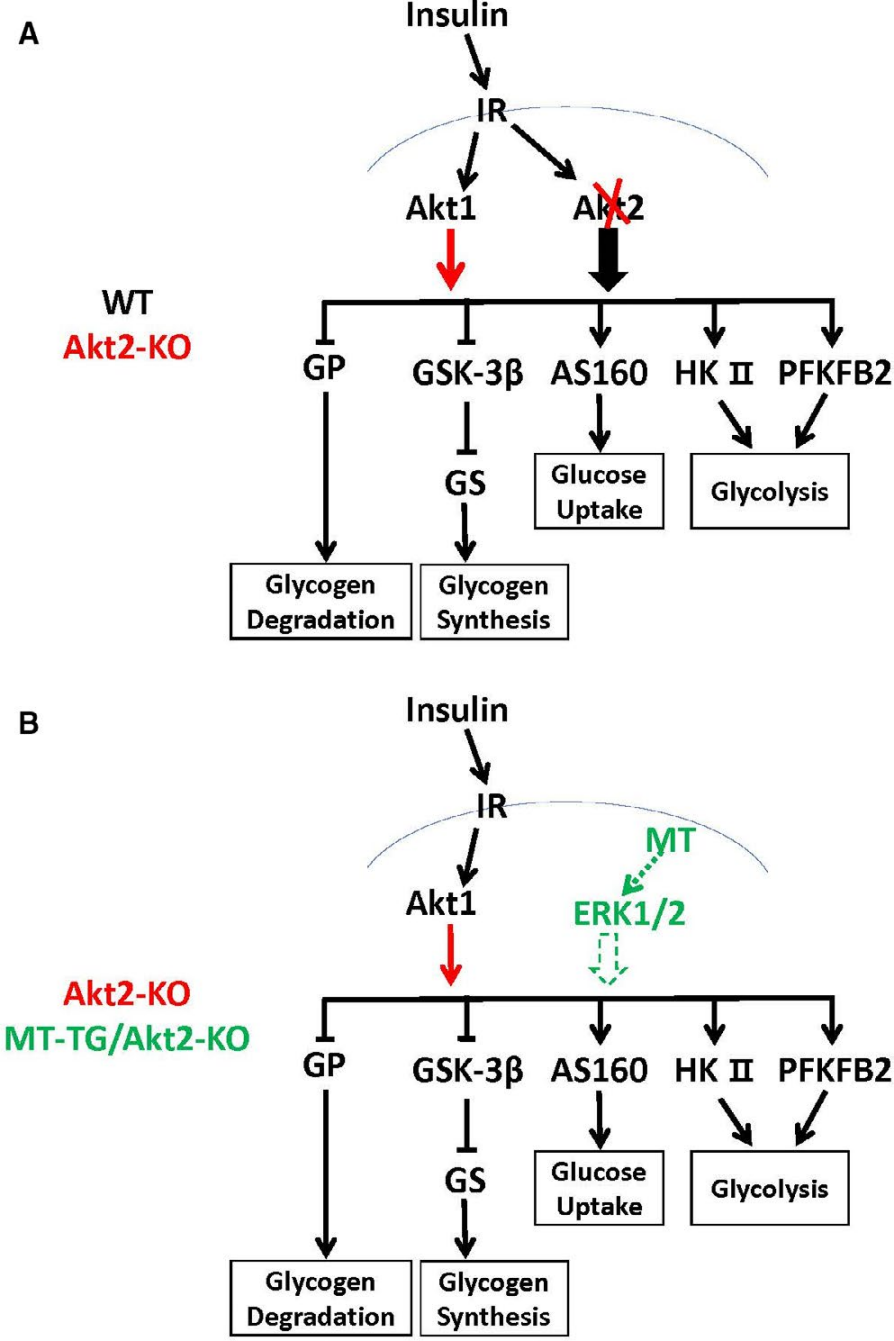
Schematic illustration of the assumed mechanisms by which the minimal glucose metabolism is compensated by up‐regulated Akt1 in the heart of Akt2‐KO mice (a) and the normal glucose metabolism is preserved by overexpressed cardiac MT in the heart of Akt2‐KO mice (b). Red line indicates the compensative changes related to glucose metabolism in the heart of Akt2‐KO mice compared to WT mice. Green lines indicate the changes related to glucose metabolism preserved by overexpressed cardiac MT in the heart of MT‐TG/Akt2‐KO mice compared to Akt2‐KO mice. Thicker arrows indicate more impact on the targets. Dashed line presents the possible pathways. IR, insulin receptor

We previously reported zinc activation of Akt2 downstream pathways related to glucose metabolism in Akt2‐KO mice, which may be associated with its inhibition of PTEN, Trb3 and PTP1B negative regulatory factors, leading to the stimulation of other isoforms of Akt phosphorylation to compensate Akt2‐KO‐associated down‐regulation of downstream gluconeogenic pathways.^18^ In the present study, phosphorylation of total Akt, probably mainly Akt1, indeed increased in the heart of Akt2‐KO mice compared with WT mice, but did not further increase in the heart of MT‐TG/Akt2‐KO mice compared to Akt2‐KO mice, and even trended lower in females (Figure 6B). These findings suggest that in Akt2‐KO or MT‐TG/Akt2‐KO mice increased Akt1 phosphorylation could compensate for other Akt2 functions but only minimally for glucose metabolic signalling to maintain glucose metabolism, therefore, resulting in typical T2D phenotype, as illustrated in Figure 8A ^11^, ^12^, ^13^, ^18^; Thus, the protective effect of cardiac overexpression of MT in MT‐TG/Akt2‐KO mice cannot be attributed to MT's stimulation of Akt1 function to preserve the almost normal level of glucose metabolism‐associated pathways, as illustrated in Figure 8B.

There must be other mechanisms by which MT preserves the glucose metabolism‐related functions and restores antioxidant functions independently on Akt2. We previously reported that renal Akt and ERK1/2 phosphorylation in MT‐KO mice was lower than that in WT mice in response to intermittent hypoxia.^32^ Phosphorylation of ERK1/2 is significantly increased after increasing MT expression.^33^, ^34^ ERKs proteins are members of the MAPK family and play important roles in various cellular events such as proliferation, differentiation, apoptosis and autophagy.^35^, ^36^ Multiple studies have described crosstalk interactions between insulin‐stimulated Akt and ERK pathways with both cross‐stimulation and cross‐inhibition but also potential synergism.^37^, ^38^ Insulin binding to IRS1 receptor stimulates the MAPK signalling cascade via Shc interactions with the Grb2‐SOS complex, followed by the stepwise activation of Ras, Raf, MEK and extracellular signal‐regulated kinase (ERK).^38^ Components of the MAPK/ERK pathway were identified as modifiers of cellular insulin responsiveness in drosophila.^39^ ERK inhibition can result in insulin resistance by down‐regulating insulin‐like receptor gene expression and ERK‐mediated physiological adjustment of insulin sensitivity allows maintenance of glucose homeostasis.^39^ ERKs are also involved in glucose metabolism: for instance, GSK‐3β direct regulation by ERK1/2 has been reported ^40^; ERK1/2 is also involved in the regulation of glucose transport ^41^, ^42^, ^43^ and glycolysis pathway.^44^, ^45^, ^46^ In the present study, there was no significant change of cardiac ERK1/2 phosphorylation in Akt2‐KO mice, but increased cardiac ERK1/2 phosphorylation in the MT‐TG/Akt2‐KO mice particularly in male mice, but significantly increased cardiac ERK1/2 phosphorylation in the MT‐TG/Akt2‐KO mice. In support of this assumption, zinc stimulating ERK1/2 function under different conditions was also reported.^47^, ^48^ Therefore, it may be possible for cardiac overexpressed MT to rescue the cardiac pathological and functional changes caused by deleting Akt2 gene via its direct or indirect activation of ERK function. The activated ERK1/2 could then stimulates glucose metabolisms via GSK‐3β and GS,^49^, ^50^, ^51^ glucose transport^52^ and glycolysis,^53^, ^54^ as illustrated in Figure 8B.

The present study confirms that MT mitigates the extent of damage caused by Akt2 deficiency similarly to our previous report showing the rescuing effect of zinc supplementation on the development of DCM in Akt2 deficient mice.^18^ In addition, numerous experiments also showed that zinc supplementation can decrease blood glucose and blood lipids in diabetic patients.^55^, ^56^, ^57^ Therefore, taken together, our results show that dietary supplemental zinc that is clinically used for other diseases may become an efficient therapy for patients with Akt2 gene mutation showing T2D symptom,^11^, ^12^, ^13^ and prevent the development of DCM.

The limitation of this study is that no cellular experiments were performed to study the pathway alone, and animal experiments were not compared at different time points to determine the specific time period at which MT protection against Akt2 deletion was achieved. In addition, the role of ERKs activation has not been evaluated by gain or loss of function experiments. These will be evaluated in future studies. However, the present study shows for the first time that cardiac overexpressing MT can restore down‐regulated glucose metabolism pathways caused by deletion of Akt2 gene, by stimulation of ERK1/2 activation in the heart to compensate for the lack of Akt2‐mediated metabolic pathways. These results may help develop therapeutic options to prevent DCM in Akt2 deficiency patients.^13^ Adequate zinc supplementation for these patients may rescue Akt2 deficiency‐caused pathogenic abnormalities.

## CONFLICT OF INTEREST

The authors declare no conflict of interest.

## AUTHOR CONTRIBUTIONS

**Shan Huang:** Conceptualization (equal); data curation (lead); formal analysis (lead); methodology (lead); project administration (equal); software (equal); validation (equal); writing‐original draft (lead); writing‐review and editing (equal). **Jiqun Wang:** Data curation (supporting); formal analysis (supporting); methodology (supporting). **Hongbo Men:** Data curation (supporting); formal analysis (supporting); methodology (equal). **Yi Tan:** Writing‐review and editing (supporting). **Qian Lin:** Data curation (supporting); formal analysis (supporting); methodology (supporting). **Evelyne Gozal:** Writing‐review and editing (equal). **Yang Zheng:** Conceptualization (lead); funding acquisition (lead); writing‐review and editing (equal). **Lu Cai:** Conceptualization (lead); formal analysis (equal); funding acquisition (equal); resources (equal); writing‐review and editing (equal).

## Supporting information

Fig S1‐S8

## Data Availability

The data that support the findings of this study are available from the corresponding author upon reasonable request.
