# Supplementary material for: Cardiac metallothionein overexpression rescues diabetic cardiomyopathy in Akt2‐knockout mice
Source: J Cell Mol Med. 2021 May 30;25(14):6828–40. doi: 10.1111/jcmm.16687 (PMC8278119; doi:10.1111/jcmm.16687)

Cardiac metallothionein overexpression rescues diabetic cardiomyopathy in Akt2-knockout mice

Shan Huang [1,2], Jiqun Wang [1,2], Hongbo Men [1,2], Yi Tan [1,3], Qian Lin [1], Evelyne Gozal [1,3], Yang Zheng [2,*], Lu Cai [1,3,4,*]

1 Pediatric Research Institute, Department of Pediatrics, University of Louisville School of Medicine, Louisville, KY 40202, USA;

2 Department of Cardiovascular Disease, The First Hospital of Jilin University, Changchun 130021, China;

3 Department of Pharmacology and Toxicology, University of Louisville, Louisville, KY 40202, USA

4 Department of Radiation Oncology, University of Louisville School of Medicine, Louisville, KY 40202, USA

***Address for correspondence**

Dr. Yang Zheng, Department of Cardiovascular Disease, First Hospital of Jilin University, Jilin University, Changchun, Jilin, 130021, China, Tel.: +86-0431-88782217; E-mail: zhengyang@jlu.edu.cn

Dr. Lu Cai, Pediatric Research Institute, Department of Pediatrics, University of Louisville, Louisville, KY, 40202, USA, Tel:502.852-2214; E-mail: lu.cai@louisville.edu

supplementary figures

Fig. S1. The original gels and detail analysis for Fig. 1C

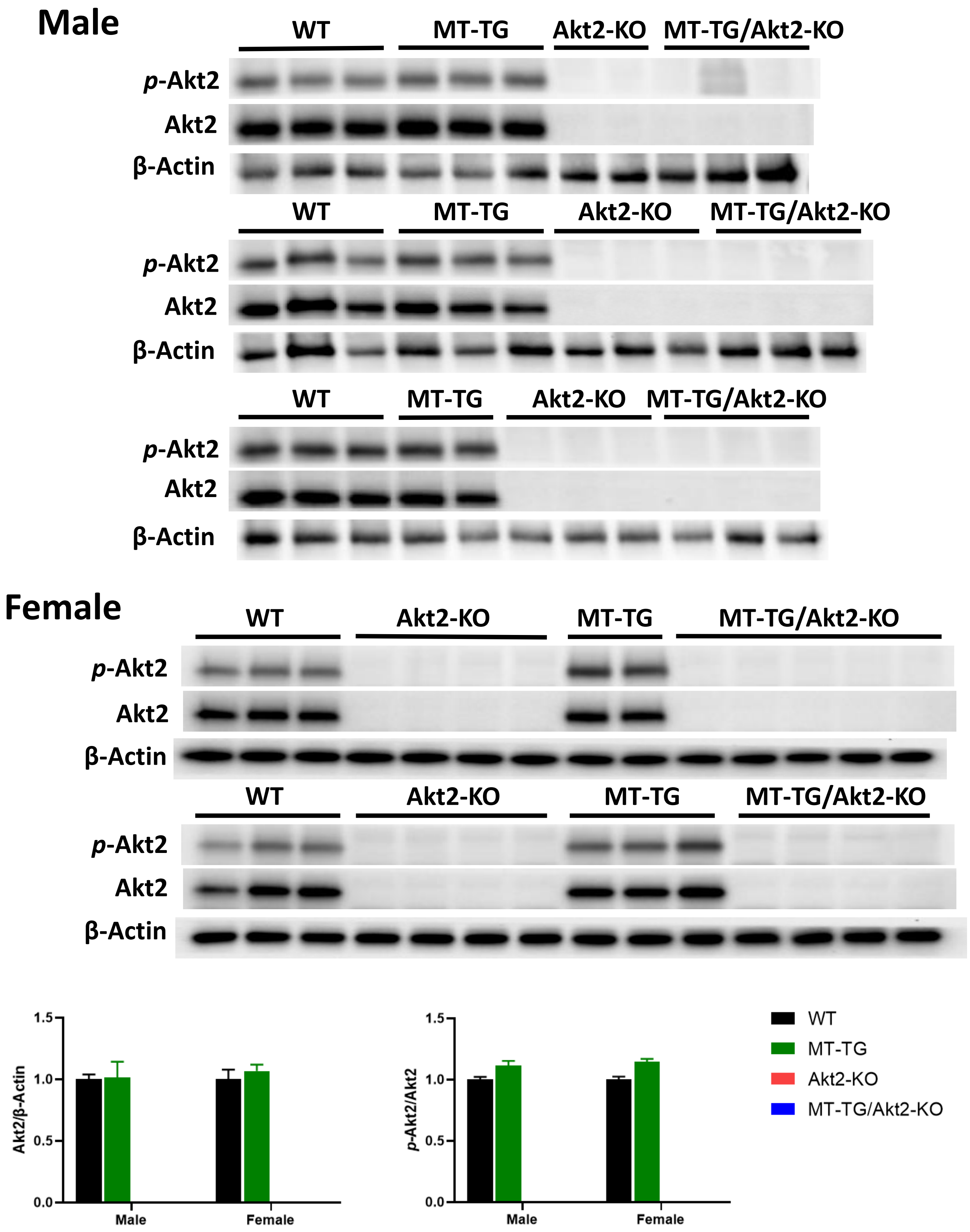

Fig. S2. The original gels and detail analysis for Fig. 6A

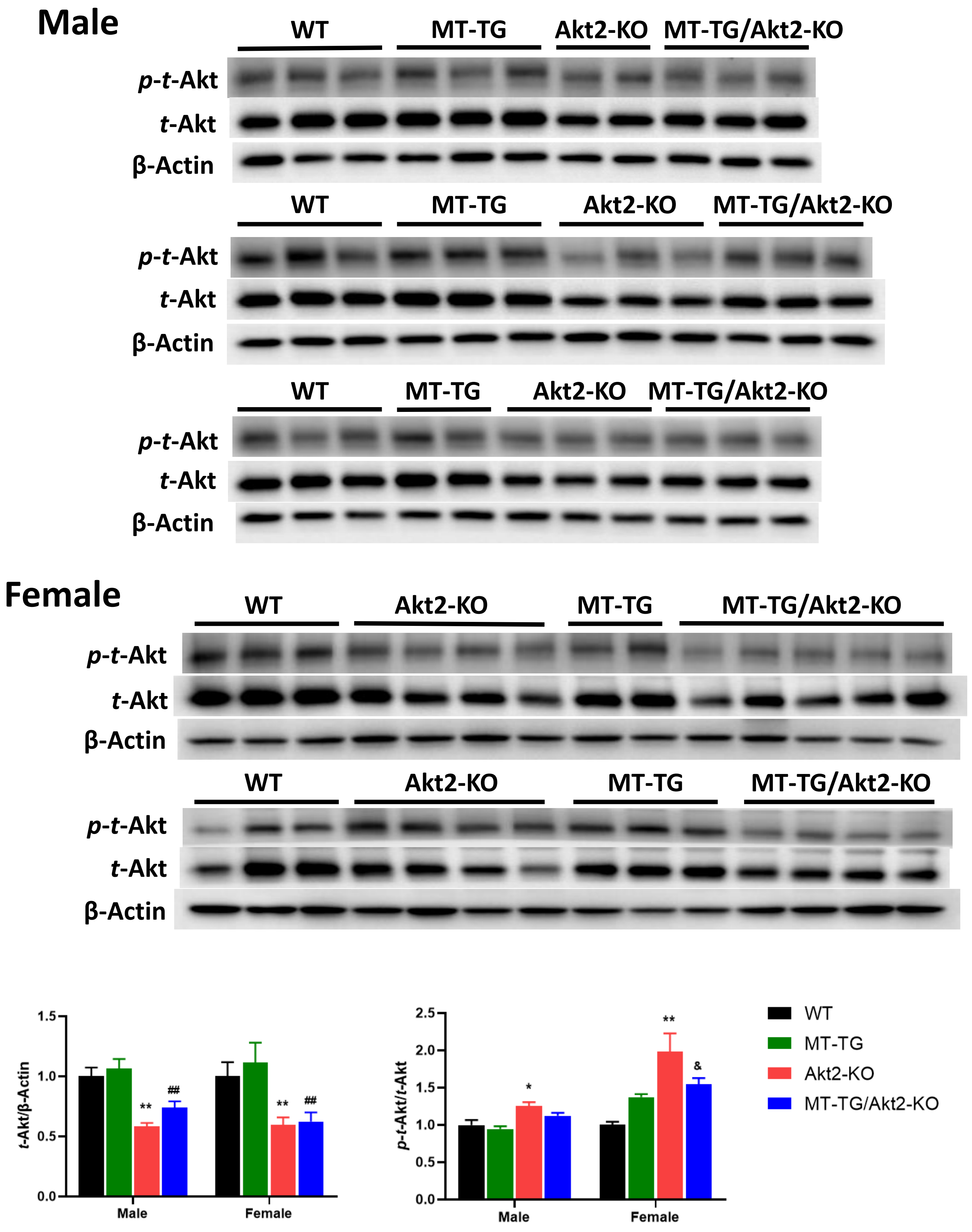

Fig. S3. The original gels and detail analysis for Fig. 6B

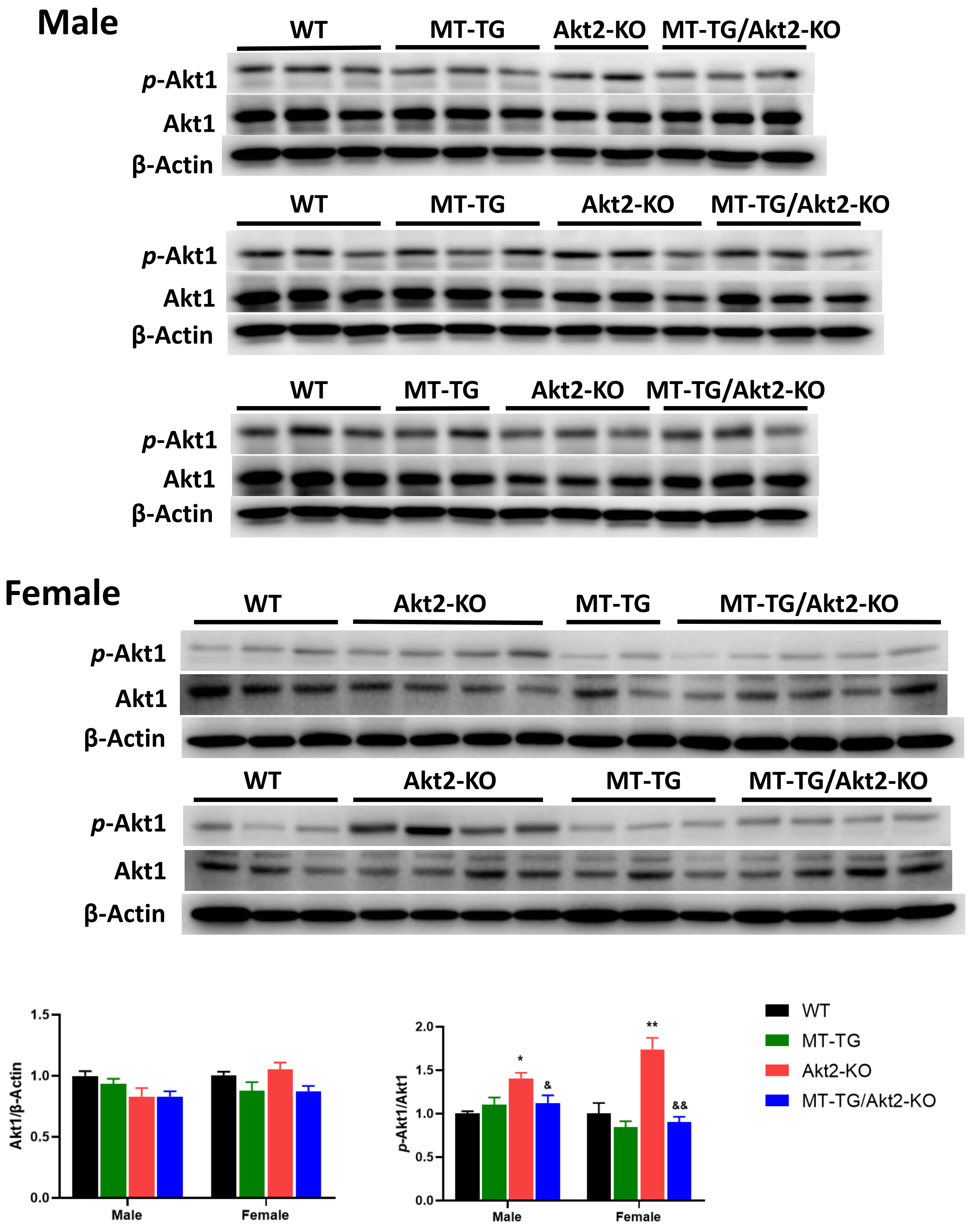

Fig. S4. The original gels and detail analysis for Fig. 6C

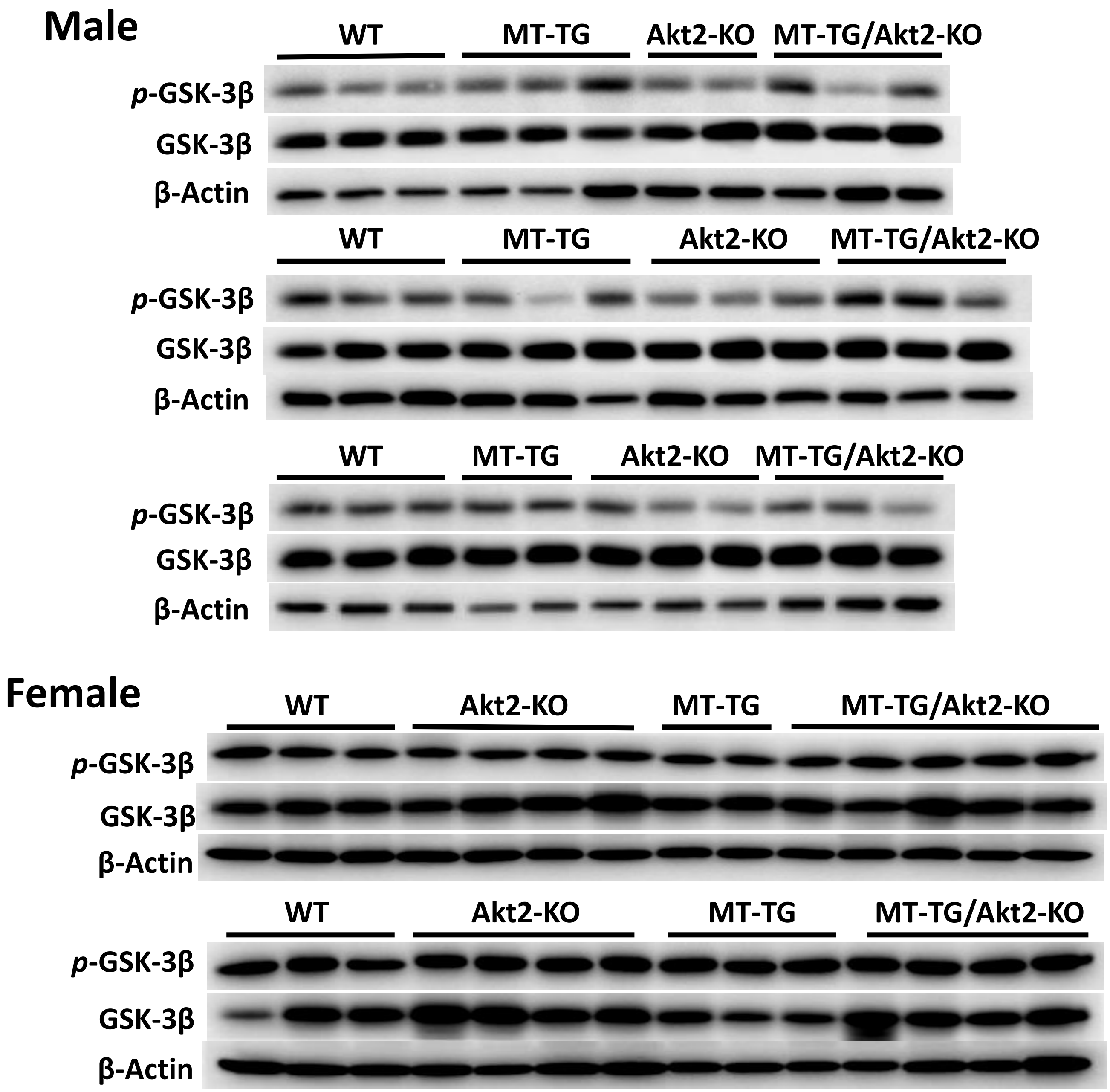

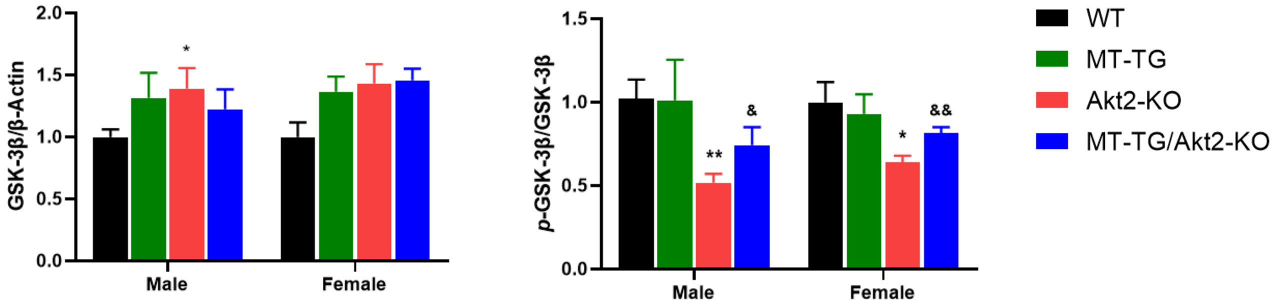

Fig. S5. The original gels and detail analysis for Fig. 6D

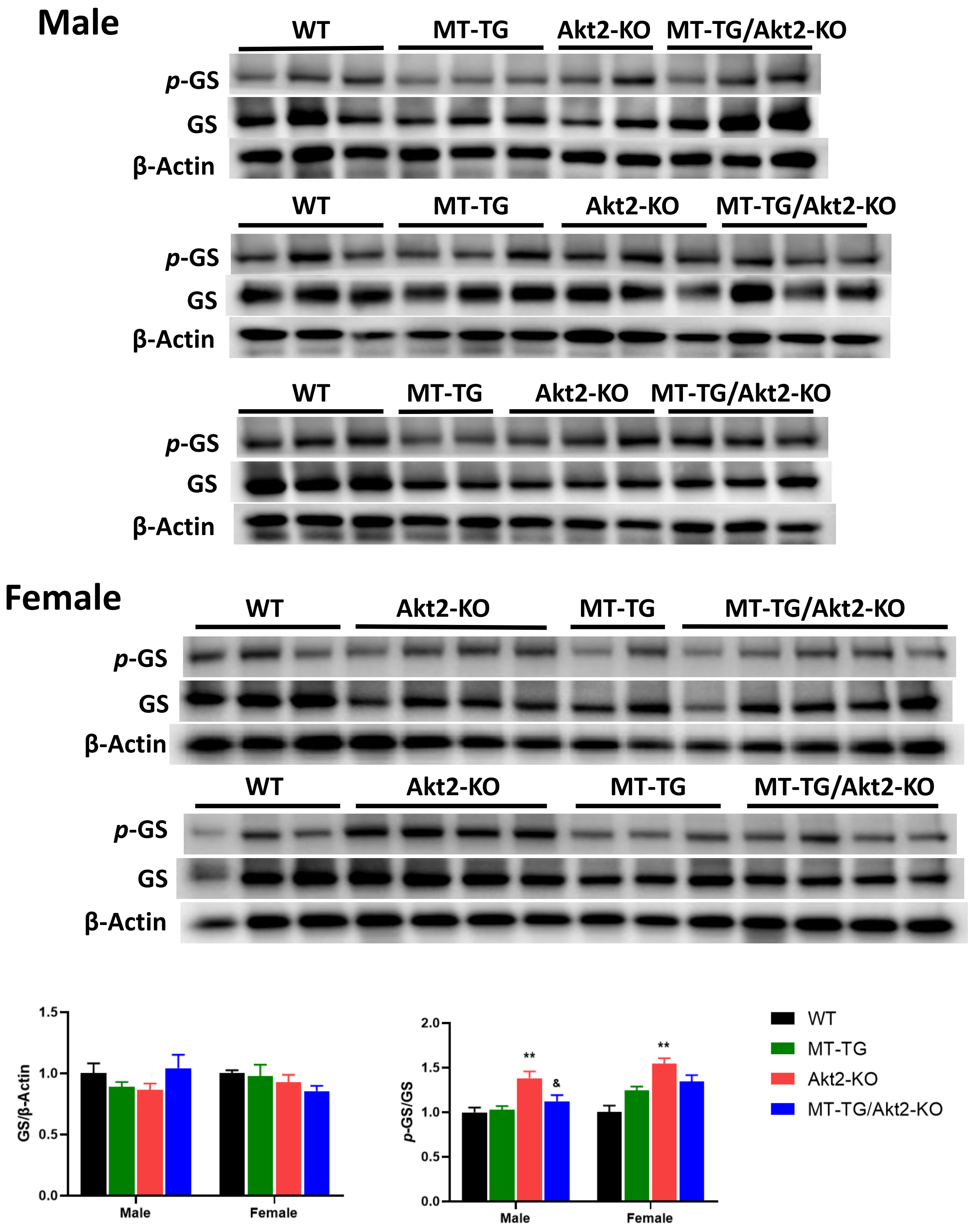

Fig. S6. The original gels and detail analysis for Fig. 7B

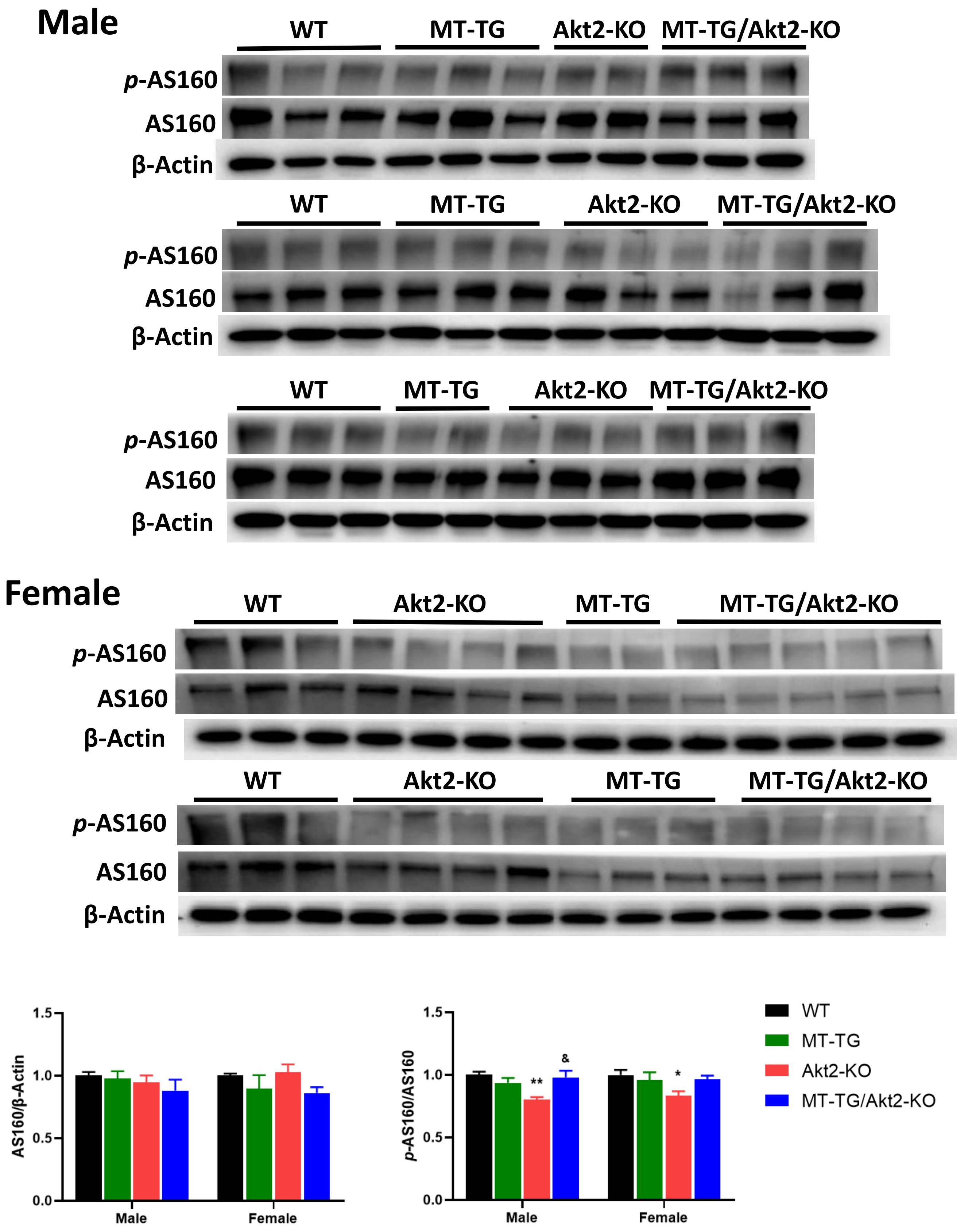

Fig. S7. The original gels and detail analysis for Fig. 7D

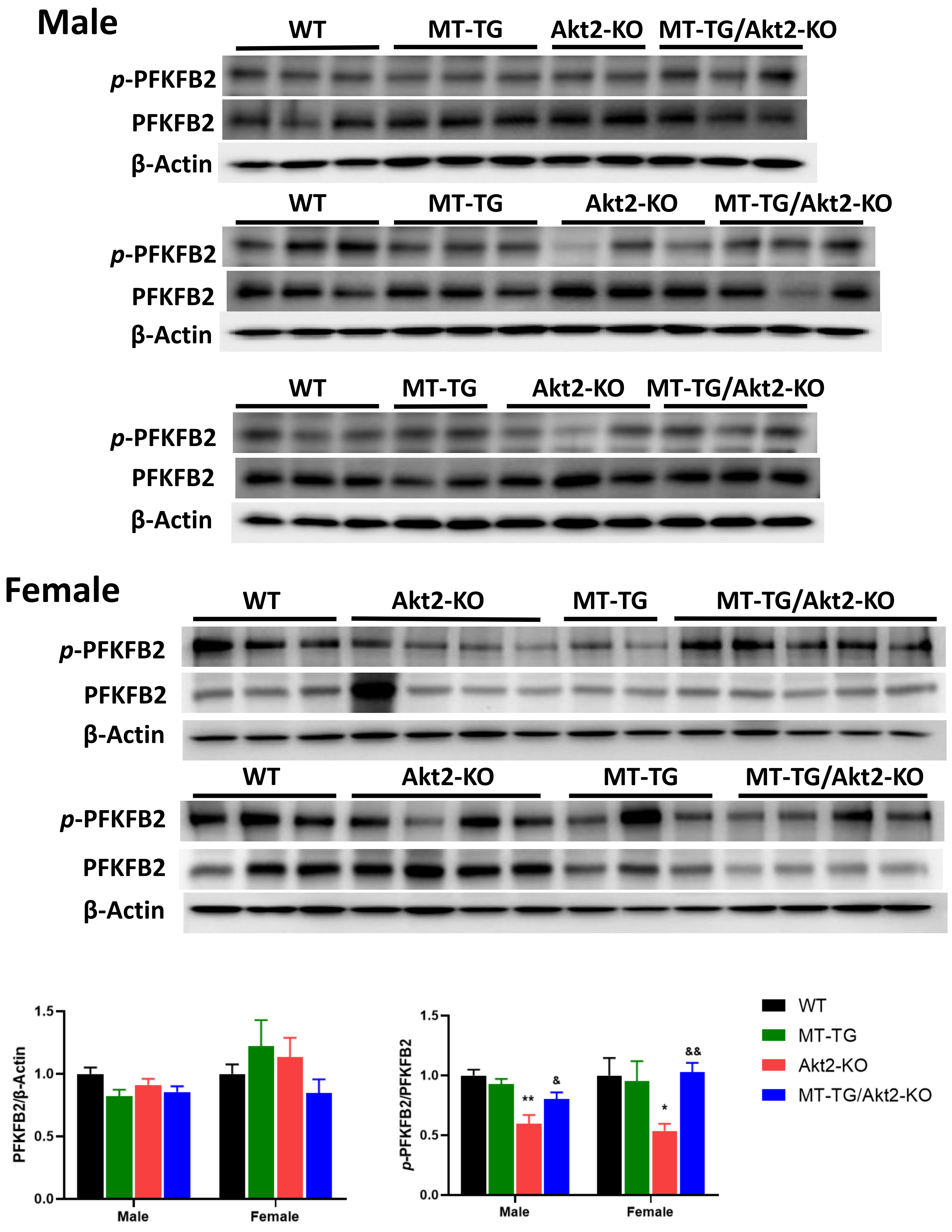

Fig. S8. The original gels and detail analysis for Fig. 7E

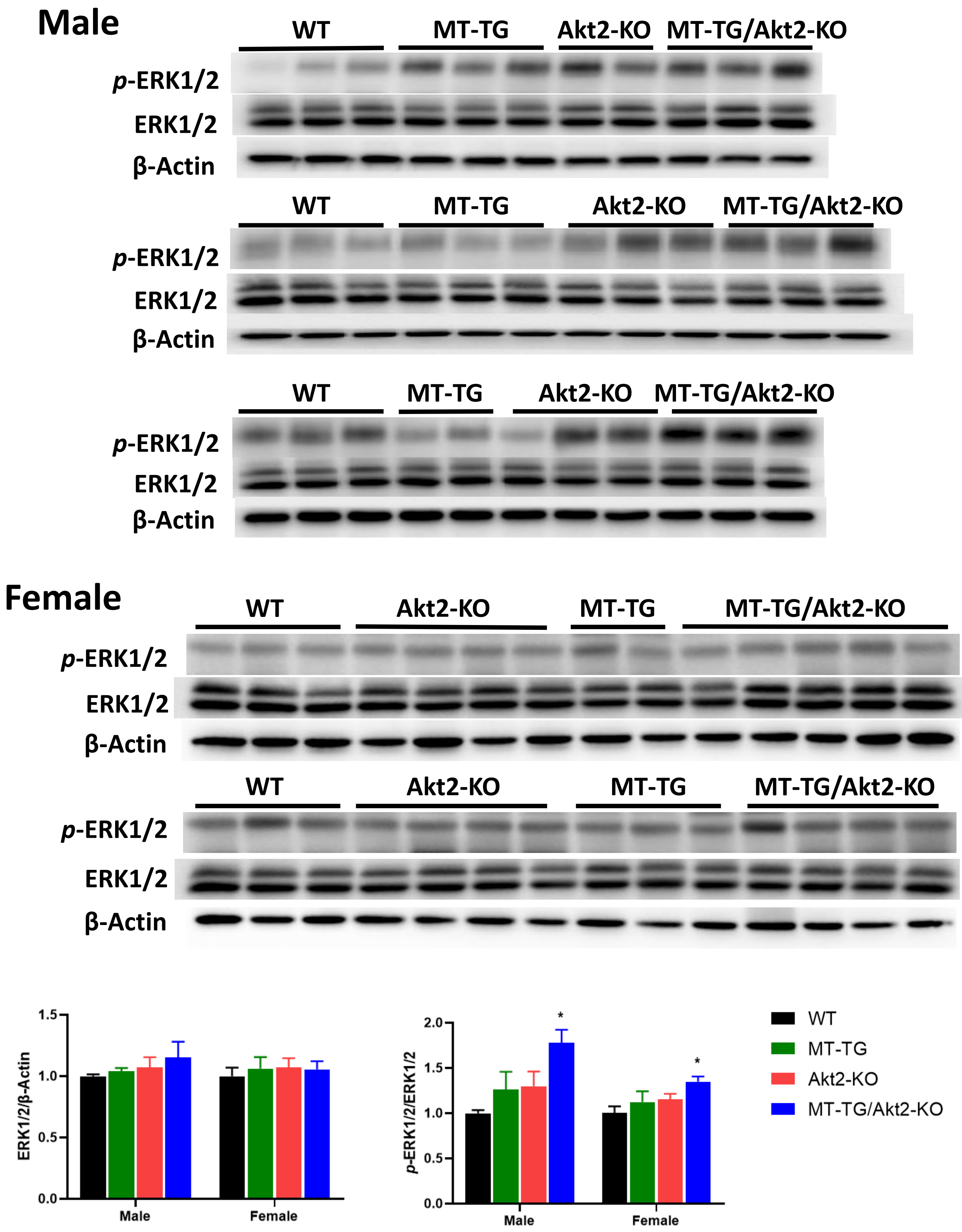

Supplement: Supplementary file 1 — Fig S1‐S8 [file JCMM-25-6828-s001.pdf]
